# Designing a multi-epitope vaccine against coxsackievirus B based on immunoinformatics approaches

**DOI:** 10.3389/fimmu.2022.933594

**Published:** 2022-11-09

**Authors:** Sichao Huang, Congcong Zhang, Jianing Li, Zongmao Dai, Jingjing Huang, Fengzhen Deng, Xumeng Wang, Xinxin Yue, Xinnan Hu, Yuxuan Li, Yushu Deng, Yanhang Wang, Wenran Zhao, Zhaohua Zhong, Yan Wang

**Affiliations:** ^1^ Department of Microbiology, Harbin Medical University, Harbin, China; ^2^ College of Bioinformatics Science and Technology, Harbin Medical University, Harbin, China; ^3^ Department of Cell Biology, Harbin Medical University, Harbin, China

**Keywords:** coxsackievirus B, viral myocarditis, immunoinformatics, epitope prediction, multi-epitope vaccine

## Abstract

Coxsackievirus B (CVB) is one of the major viral pathogens of human myocarditis and cardiomyopathy without any effective preventive measures; therefore, it is necessary to develop a safe and efficacious vaccine against CVB. Immunoinformatics methods are both economical and convenient as *in-silico* simulations can shorten the development time. Herein, we design a novel multi-epitope vaccine for the prevention of CVB by using immunoinformatics methods. With the help of advanced immunoinformatics approaches, we predicted different B-cell, cytotoxic T lymphocyte (CTL), and helper T lymphocyte (HTL) epitopes, respectively. Subsequently, we constructed the multi-epitope vaccine by fusing all conserved epitopes with appropriate linkers and adjuvants. The final vaccine was found to be antigenic, non-allergenic, and stable. The 3D structure of the vaccine was then predicted, refined, and evaluated. Molecular docking and dynamics simulation were performed to reveal the interactions between the vaccine with the immune receptors MHC-I, MHC-II, TLR3, and TLR4. Finally, to ensure the complete expression of the vaccine protein, the sequence of the designed vaccine was optimized and further performed *in-silico* cloning. In conclusion, the molecule designed in this study could be considered a potential vaccine against CVB infection and needed further experiments to evaluate its safety and efficacy.

## Introduction

Viral myocarditis (VMC), an inflammatory disease of the myocardium resulting from a viral infection, represents the main cause of sudden cardiac death and heart failure in adolescents (1, 2). More than 20 viruses have been associated with VMC in humans (3). However, coxsackievirus B (CVB) is recognized as the major pathogen of VMC (4–6).

CVB is a positive single-stranded RNA (ssRNA) virus, which belongs to the *Enterovirus* genus within the *Picornaviridae* (7, 8). As with other members of *Picornaviridae*, the virion of CVB is a 30-nm non-enveloped icosahedral particle. Six serotypes have been identified (CVB1–CVB6). The genome of CVB is approximately 7.4 kb in size and encodes four structural proteins (VP1, VP2, VP3, and VP4) and seven non-structural proteins (2A, 2B, 2C, 3A, 3B, 3C, and 3D) (3, 8). Capsid proteins VP1, VP2, and VP3 are exposed at the virion surface, whereas VP4 is located inside and linked to the viral RNA (9). Among the four structural proteins, VP1 is the most external capsid protein and exhibits a high-sequence variability (10). The epitopes that bind neutralizing antibodies are mostly located at VP1, which is thus considered a potential vaccine candidate. Only a few epitopes are distributed in VP2 and VP3 (11). In addition, the non-structural proteins play very diverse but specialized roles in the replication of CVB. They facilitate viral protein synthesis, replication, release, and dissemination by interacting with RNA genomes and polyproteins, while they also participate in interfering with various cellular processes (12, 13).

At present, vaccination is the most effective intervention against virus infection (14). A variety of vaccines against CVB have been developed, including inactivated or attenuated vaccines, RNA vaccines, DNA vaccines, recombinant protein vaccines, and virus-like particle vaccines (15–19). Nevertheless, there are still no approved vaccines or antiviral drugs available for clinical use against CVB infection. Compared with the traditional vaccine, the multi-epitope vaccine is a novel strategy of vaccine development in recent years, which can directly induce a specific CD4^+^ and CD8^+^ T-cell immune response against the chosen epitopes and avoid the side effects of other adverse epitopes in the intact antigen (20, 21). The potential advantage of an epitope-based vaccine includes safety and stability. The problems of traditional vaccines including virus excretion and virulence recovery may also be properly addressed (22). Therefore, we tried to develop a novel multi-epitope vaccine against CVB.

Vaccine development based on conventional methods is very laborious and complicated (23). Immunoinformatics provides an *in-silico* approach that facilitates the rapid development of a potential multi-epitope vaccine in a short time and is recognized as an efficient and economical approach than the traditional procedure under laboratory conditions (24–26). There are attempts to design multi-epitope vaccines based on viral structural and non-structural proteins by using immunoinformatics tools. Alam et al. proposed an efficient strategy for designing multi-epitope vaccines against different viruses, including the Zika virus and dengue virus, as well as SARS-CoV-2 (27–31). In this study, we utilized immunoinformatics tools to analyze the conserved epitopes of CVB proteins and developed a multi-epitope vaccine. These proteins included VP1, VP2, VP3, 2A, 2C, and 3C. Then, the vaccine was evaluated by a series of immunoinformatics methods to verify its stability and efficacy. It was identified that the multi-epitope vaccine designed in this study could make strong interactions with human immune receptors and induce a robust host immune reaction.

## Materials and methods

### Retrieving protein sequences

The complete amino acid sequences of CVB (AAA42931.1, AAC00531.1, AUF49670.1, AAL37156.1, QAT18823.1, and ALA40024.1) were retrieved from the NCBI Protein database (https://www.ncbi.nlm.nih.gov/protein) as the vaccine candidates and sorted in FASTA format (32–35). The protein components include four structural proteins (VP1, VP2, VP3, and VP4) and seven non-structural proteins (2A, 2B, 2C, 3A, 3B, 3C, and 3D). The proteins with <100 amino-acid sequences that were too short to analyze epitopes were removed, and the rest of the proteins were utilized to predict antigenicity.

### Protein antigenicity prediction

The antigenicity of the identified proteins was predicted by VaxiJen v2.0 and ANTIGENPro. VaxiJen v2.0 is based on auto cross covariance (ACC) transformation of protein sequences into uniform vectors of principal amino acid properties. The method shows a prediction accuracy ranging from 70% to 89% (36). ANTIGENPro is a convenient online server that utilized specific microarray data for the calculation of protein antigenicity scores (37). Proteins with an antigenic score ≥0.4 were considered to have antigenicity and were selected for further predicted epitopes.

### Cytotoxic T lymphocyte epitope prediction

For designing a subunit vaccine, it is important to accurately predict the cytotoxic T lymphocyte (CTL) epitopes. NetCTL-1.2, a high-sensitivity approach based on artificial neural networks, was used to predict the CTL epitopes from selected proteins (38). For CTL epitope prediction, a default threshold value of 0.75 and three different supertypes (A2, A3, and B7) were selected for epitope identification (39). The CTL epitopes were selected depending on the combined score and half-maximal inhibitory concentration (IC_50_) <50 nm (40–42). According to the criteria, high scores indicated favorable binding, and IC_50_ <50 nm represents the epitope having the best affinity to the receptor. NetMHCpan 4.1, a reliable method for predicting the binding of peptides to MHC molecules of any known sequence, was employed to check the prediction results from NetCTL-1.2 (43).

### Helper T lymphocyte epitope prediction

The helper T lymphocyte (HTL) epitopes for the selected proteins were determined by the NetMHCII-2.3 and NetMHCIIpan 4.0 servers (44, 45). We set the peptide length of epitopes as 15 mer and selected 14 alleles of human leukocyte antigen (HLA) for the HTL epitope prediction (46). The HTL epitopes were selected according to IC_50_ <50 nm and least percentile ranks. The epitopes that exhibited recurrence in both tools were selected as candidate epitopes and prepared for further analysis.

### IFN-γ inducing epitope prediction

IFN-γ is induced by antigenic stimuli and plays a vital role in both innate and adaptive immunity by stimulating macrophages and natural killer cells (47). IFN-γ not only possesses a broad-spectrum antiviral activity but also heightens the response of MHC to antigens (48). The IFN-γ epitope server was employed to identify IFN-γ epitopes with the hybrid approach based on a support vector machine (SVM) (49). The chosen HTL epitopes were entered into the IFN-γ epitope server, and only the IFN-γ-inducing epitopes were selected for the final vaccine construction.

### Linear and conformational B-cell epitope prediction

The linear B-cell epitopes were predicted *via* the ABCpred and BCPreds online servers (50, 51). The threshold and window length to use for prediction were set at 0.75 and 16 mer, respectively. The overlapping filter was kept during the epitope prediction. Finally, only epitopes that overlap between the ABCpred and BCPreds servers were chosen as candidate epitopes for further analysis.

ElliPro, a reliable online server based on the 3D structure of the protein, was utilized to predict conformational (discontinuous) B-cell epitopes in the present study (52). ElliPro associates each discontinuous epitope with a protrusion index (PI) value averaged over epitope residues. Conformational B-cell epitopes with a PI value >0.9 were selected for further confirmation. The DiscoTope server, based on solvent-accessible surface area calculations and contact distances to predict discontinuous B-cell epitopes, was used to confirm the prediction result from ElliPro (53).

### Conservancy evaluation

The CLUSTALW server was used to align the multiple CVB sequence. The results of sequence alignment were viewed by ESPript 3.0 (54). Only epitopes located in highly conserved regions were selected for vaccine construction.

### Multi-epitope subunit vaccine construction

The final vaccine sequence was devised by concatenating all conserved epitopes predicted by various immunoinformatics tools with a suitable adjuvant and linkers. The epitopes of CTL, HTL, and B cell were linked by AAY, GPGPG, and KK linkers, respectively. To increase the immunogenicity of the vaccine, the β-defensin amino acid sequence and pan-HLA DR binding epitopes were adjoined to the vaccine N-end with the help of the EAAAK linker (55, 56). The β-defensin peptides recruit naive T cells and immature dendritic cells through chemokine receptor-6 (CCR-6) and provide an adaptive immune response (55). The addition of pan-HLA DR binding epitope sequence to the multi-epitope vaccine is to facilitate binding to different types of mouse and human MHC-II alleles to trigger T-cell immune responses (56). Additionally, the TAT sequence was added to the C-terminal *via* the KK linker to promote the intracellular transport of the vaccine. Inserting the specific linker between two epitopes can make each epitope play an independent immune effect and avoid generating new epitopes that mask the original epitope, which is necessary to make sure every epitope functions effectively (57).

### Secondary structure prediction

The PSIPRED Workbench server was used to predict the secondary structure of the CVB vaccine (58). PSIPRED provides a variety of accurate protein annotation tools that allow users to easily perform truly scalable biological analysis. In this study, we submitted the complete sequence data of the designed vaccine and chose PSIPRED 4.0 as the prediction method.

### Allergenicity, antigenicity, and solubility evaluation

The allergenicity of the final vaccine was evaluated by AllerTOP v.2.0 and AllergenFP 1.0 online servers. AllerTOP is a freely accessible server for allergen prediction based on machine learning. According to the instruction, the prediction accuracy of this tool was reported to be 85.3% (59). AllergenFP described the amino acids as five E-descriptors, and the strings were transformed into uniform vectors by ACC transformation (60). The solubility of the designed vaccine was predicted *via* the SolPro and Protein-Sol servers (61, 62).

### Physicochemical properties and toxicity evaluation

The toxicity and physicochemical properties of epitopes were assessed by ToxinPred2 and ProtParam, respectively. ToxinPred2 is specifically developed for predicting the toxicity of peptides or for designing peptides with the desired toxicity (63). ProtParam is a flexible server that allows the analysis of the various physical and chemical properties of the submitted protein. The computed properties mainly include the molecular weight, theoretical isoelectric point (PI), amino acid composition, atomic composition, extinction coefficient, half-life *in vitro* and *in vivo*, instability and aliphatic indexes, and the grand average of hydropathicity (GRAVY) (64).

### Immune simulations

To record the immune response profile of the designed vaccine, *in-silico* immune simulation programs were performed by employing the C-ImmSim online server (65). The C-ImmSim model defines both the humoral and cellular responses of a mammalian immune system against vaccine construction. In this study, all simulation parameters were set as default, and the time steps of three injections were set at 1, 252, and 504, respectively.

### Tertiary structure prediction, refinement, and validation

The tertiary structure, also known as the three-dimensional (3D) structure, of the CVB vaccine was predicted by using the AlphaFold2 program (66). Subsequently, the predicted structure was refined by 50-ns all-atom molecular dynamics (MD) simulations using the AMBER20 package (67). Clustering analysis was then performed on the MD trajectory using the CPPTRAJ program to select the representative vaccine structure, i.e., the representative structure of the most populated cluster. Further evaluations were based on this selected vaccine structure.

To validate the 3D structure model of the developed vaccine protein, the Ramachandran plots of the initial model and the refinement model were generated separately *via* the SWISS-MODEL web server (68). The Ramachandran plot reflects whether the dihedral angle [psi (ψ) and phi (ϕ)] of amino acid residues in protein structure is within a reasonable range (69). In addition, ProSA-web was conducted to validate the vaccine 3D structure (70). The evaluation of the model quality is based on *Z*-score; a positive *Z*-score implies that there are unreasonable or unstable sections in the generated 3D protein model.

### Molecular docking of the CVB vaccine against the antigenic recognition receptor

An effective immune reaction can only be induced when the antigenic molecule interacts with the specific immune receptor in the host. Thus, protein–protein docking was performed to reveal the binding affinity between the vaccine protein and antigenic recognition receptors of MHC-I (PDB ID: 4WUU), MHC-II (PDB ID: 3C5J), TLR3 (PDB ID: 2A0Z), and TLR4 (PDB ID: 3FXI) by using the ClusPro2.0 server (71). ClusPro2.0 directly performs rigid docking *via* the PIPER tool, a docking program based on the fast Fourier transform (FFT) algorithms that perform exhaustive sampling of the conformational space on a dense grid, to sample the most near-native structures for more accurate docking structure. After obtaining the results from the ClusPro server, the docked complex was visualized *via* the molecular graphic program PyMOL (72). The binding energy, interface area, and hydrogen bonds were analyzed by the PDBePISA and PDBsum servers (73, 74).

### Molecular dynamic simulation

To investigate the dynamic behavior of the vaccine and receptor complex (MHC-I, MHC-II, TLR3, and TLR4), the complexes were subject to all-atom MD simulations *via* the AMBER20 package. The proteins were immersed into an OPC water box with protein atoms that were at least 12 Å away from the edge of the box. Potassium and chloride ions were added to neutralize the system’s charge. The amber ff19SB protein force field (75) and the Joung/Cheatham ion parameter (76) set were used. To ensure that the system has no steric clashes or inappropriate geometry, energy minimization was performed by 200 steps of the steepest descent method and 1,800 steps of the conjugate gradient method. The energy-minimized system was heated from 0 to 100 K in the NVT ensemble over 40 ps and from 100 to 300 K in the NPT ensemble over 200 ps. A harmonic constraint with a force constant of 5 (kcal mol^−1^ Å^−2^) was applied to the non-hydrogen atoms during the heating stage. A 400-ps equilibrium simulation was carried out on the system with the force constant reduced to 1 kcal/mol in the NPT ensemble at 300 K. The SHAKE algorithm (77) was used to restrain the length of bonds involving hydrogen. The Langevin thermostat was used during the simulations. Hydrogen mass repartition was performed to enable a 4-fs time step, and the non-bonded interaction cutoff was 10 Å. Finally, a 50-ns production MD simulation was performed *via* the pmemd program with trajectory coordinates recorded every 20 ps (78). After simulation, the trajectory generated by the MD was analyzed by the CPPTRAJ module, and calculations of the binding free energy of the simulated complexes were performed by the MMPBSA.py script from the AMBER20 package.

### Codon adaptation, mRNA secondary structure prediction, and *in-silico* cloning

To stably and effectively express the vaccine protein in the prokaryotic expression system by *Escherichia coli* (*E. coli*), the amino-acid sequence of the designed vaccine was reverse-translated and optimized by performing the Java Codon Adaptation tool (79). The *E. coli* K12 strain was utilized to transform the CVB vaccine. During optimization, the prokaryotic ribosome binding sites, restriction enzyme cleavage sites, and Rho-independent transcription termination were chosen to assure the complete translation of the vaccine gene. Then, the secondary structure of mRNA was generated *via* RNAfold tools (80). Finally, the restriction endonuclease sites *Hin*dIII and *Bam*HI were inserted into the N- and C-ends of the optimized DNA sequence for its *in-silico* cloning into the pET-28(+) vector by employing the SnapGene software.

## Results

**Figure 1:**
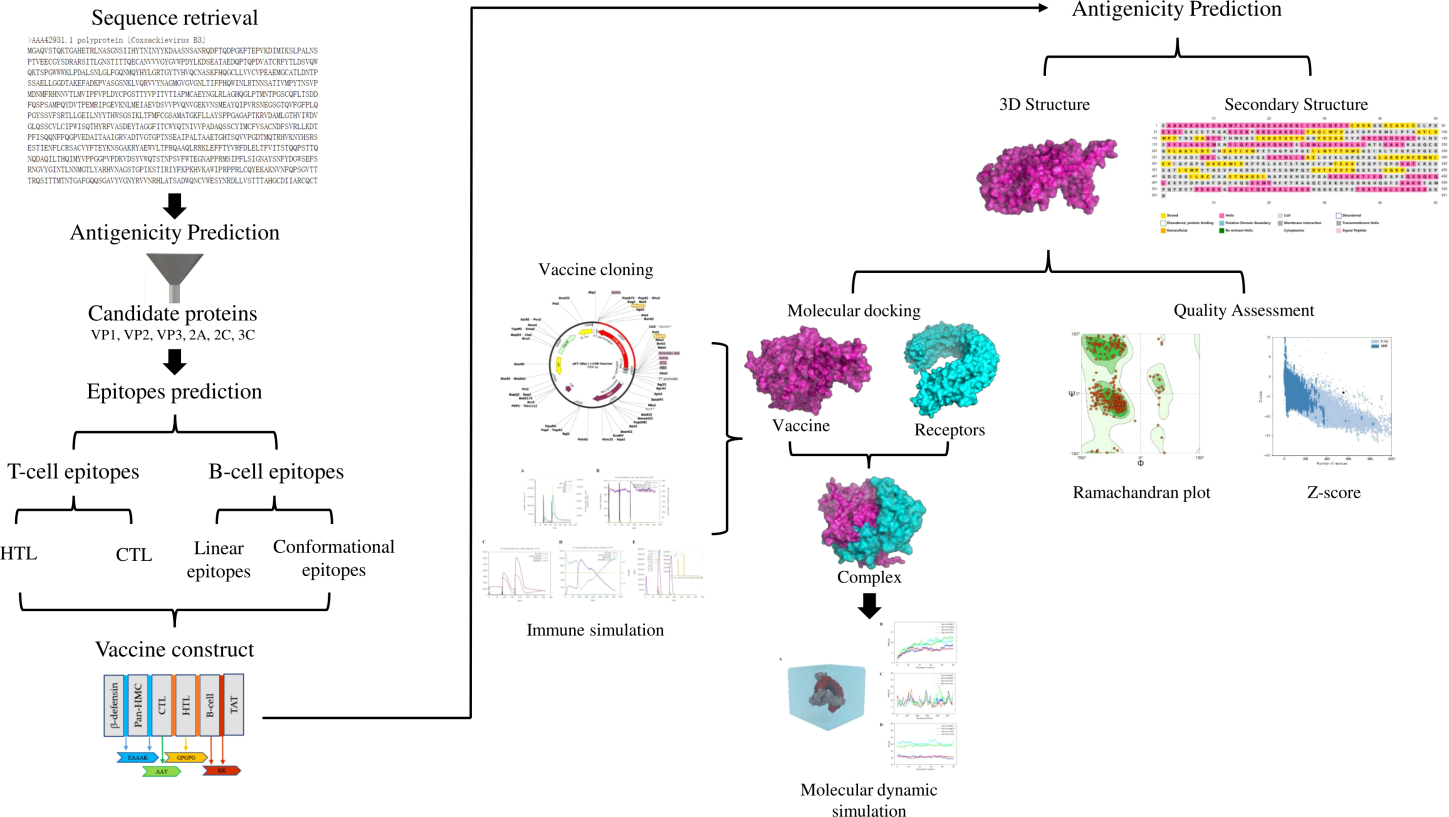
Flow diagram of the multi-epitope vaccine design in this study.

The design process of the multi-epitope vaccine against CVB is illustrated in **Figure 1**.

### Antigenicity analysis of CVB and selection of protein for vaccine development

The proteins of CVB were obtained from the NCBI Protein Database, which comprised 11 proteins. The reference sequences of those proteins were conserved in FASTA format as shown in **Table 1**. Four proteins with <100 amino-acid sequences (VP4, 2B, 3A, and 3B) that were too short to forecast epitopes were removed.

**Table 1:** Details and antigenic value of the CVB proteins.

No.	Protein	Amino acids	ANTIGENPro score	VaxiJen score
1	VP1	284 aa	0.9704	0.6305
2	VP2	263 aa	0.8708	0.5678
3	VP3	238 aa	0.7833	0.4120
4	VP4	69 aa	0.6492	0.3219
5	2A	147 aa	0.6033	0.5498
6	2B	99 aa	0.5867	0.2231
7	2C	329 aa	0.7720	0.5359
8	3A	89 aa	0.3277	0.4146
9	3B	22 aa	0.5256	0.3263
10	3C	183 aa	0.5896	0.5959
11	3D	462 aa	0.5936	0.4692

For the development of an effective epitope vaccine, it is significant to identify candidate proteins that can elicit a certain protective immune response. The sequences of the remaining seven proteins were submitted to VaxiJen and ANTIGENPro to confirm their antigenicity according to the antigenic score (**Table 1**). Proteins with an antigenic score ≥0.4 were considered to have high antigenicity. Finally, we selected the proteins VP1, VP2, VP3, 2A, 2C, and 3C according to their functions in the process of viral infection to predict epitopes.

### Cytotoxic T lymphocyte epitope prediction

The CTL (9 mer) epitopes of the selected protein were analyzed through the NetCTL and checked by the NetMHCpan server. The CTL epitope was selected according to the highest combined score and an IC_50_ value <50 nm. Among these epitopes, 20 were selected as the candidates for vaccine development, as illustrated in **Table 2**.

**Table 2:** Predicted cytotoxic T lymphocyte (CTL) epitopes of CVB proteins utilized for the construction of a novel multi-epitope vaccine.

Protein	CTL epitopes predicted by using the NetCTL server		
	A2 supertype (IC_50_)	A3 supertype (IC_50_)	B7 supertype (IC_50_)
VP1	ILTHQIMYV (15.15)	KSTIRIYFK (37.71)	APPRMSIPF (47.28)
VP2	IVMPYTNSV (13.02)	KTSPGWWWK (48.79)	MPYTNSVPM (6.84)
VP3	RLLKDTPFI (7.27)	YTHWSGSIK (89.14)	APTKRVDAM (8.67)
2A	AVYVGNYRV (85.83)		YPRRYQSHV (24.54)
2C	KLNSSVYSL (6.44)	KMSNYIQFK (7.92)	QVRYSLDML (149.51)
3C	FLAKEEVEV (7.36)	AVLAINTSK (61.24)	RAGQCGGVL (79.83)

### Helper T lymphocyte epitope prediction

The HTL epitopes (15 mer) were predicted by the NetMHCII server for the three most common HLA supertypes (HLA-DR, HLA-DQ, and HLA-DP) (29). The potential epitopes were selected with the lowest scores (the lowest scores represented the highest binding capability of epitopes) and an IC_50_ value <50 nm. Then, the epitopes predicted by NetMHCII were validated by the NetMHCIIpan server. Only the epitopes that showed recurrence in both tools were further screened based on positive IFN-γ induction. Finally, 15 HTL epitopes were chosen for subsequent analysis (**Table 3**).

**Table 3:** Predicted helper T lymphocyte (HTL) epitopes of CVB proteins utilized for the construction of a multi-epitope vaccine.

Epitope	Allele	IC_50_
VP1		
KLEFFTYVRFDLELT	HLA-DPA1^*^01:03/DPB1*04:01	5.7
LEFFTYVRFDLELTF	HLA-DPA1^*^03:01/DPB1*04:02	15.4
VP2		
VQRVVYNAGMGVGVG	HLA-DRB1^*^09:01	16.9
GNLTIFPHQWINLRT	HLA-DRB4^*^01:01	29.3
LRTNNSATIVMPYTN	HLA-DQA1^*^01:02/DQB1^*^06:02	20.8
VP3		
EILNYYTHWSGSIKL	HLA-DRB1^*^07:01	4.9
LNYYTHWSGSIKLTF	HLA-DRB1^*^09:01	25
2A		
EGVVGFADIRDLLWL	HLA-DQA1^*^01:01/DQB1^*^05:01	32.4
2C		
RKYAPLYAAEAKRVF	HLA-DRB1^*^01:01	3.3
SVATNLIGRSLAEKL	HLA-DRB1^*^07:01	13.7
CRKYAPLYAAEAKRV	HLA-DRB1^*^09:01	20.4
ALARRFHFDMNIEVI	HLA-DQA1^*^01:01/DQB1*05:01	18.5
KFIEWLKVKILPEVR	HLA-DPA1^*^02:01/DPB1*01:01	36.1
3C		

### Linear and conformational B-cell epitope prediction

The ABCpred and BCPreds servers were employed to identify the linear B-cell epitopes. All predicted epitopes (16 mer) with a prediction score >0.75 and which overlapped in both servers were selected. Among these line epitopes, 29 epitopes were selected as the candidate epitopes. (**Table 4**). The discontinuous B-cell epitopes were predicted by using the ElliPro server and checked by the DiscoTope server. Finally, a total number of 15 discontinuous B-cell epitopes were chosen for further analysis (**Table 5**).

**Table 4:** Predicted line B-cell epitopes of CVB proteins utilized for the construction of a multi-epitope subunit vaccine.

Protein	Sequence	Start position
VP1	QQPSTTQNQ	124
	GGPVPDKVDSY	147
	TSTNPSVFWTE	161
	GSTGPIKST	224
	HVKAWIPRPPRL	241
	TTTRQSITTMTNTGA	269
VP2	GYGVWPDYLKDS	34
	EDQPTQPDVATCR	50
	VVCVPEAEMGCAT	124
	TAKEFADKPVAS	151
	SATIVMPYTNSVP	199
	MVIPFVPLDYCPGSTT	224
VP3	DDFQSPSAMPQYDVT	17
	EVDSVVPVQNVGE	48
	NEGSGTQVFGFPLQP	75
	PGAGAPTKRVDA	133
	FVASDEYTAGGFIT	178
2A	QESEYYPKRYQSH	82
	AGFSEPGDCGGILRC	99
	VTMGGEGVVG	121
2C	LPEVREKHEFLN	37
	TIEQSAPSQSDQEQLF	62
	HGSPGAGKSV	128
	PPDPDHFDGYKQQ	158
	ASTNAGSINAP	220
3C	EVEVNEAVL	94
	MYNFPTRAGQCG	137
	HVGGNGHQGFSAA	161

**Table 5:** Predicted conformational B-cell epitopes of CVB proteins.

Protein	Residues	Number of residues
VP1		
1	S127, T128, T129, Q130, N131	5
2	T271, R272, Q273, S274, I275, T276, T277, M278, T279, N280, T281	11
3	T20, G21, P22, T23, N24, S25, E26, A27, M48, Q49, T50	11
4	W196, E198, S200, R201, N202, G203	6
VP2		
VP3		
1	S23, A24, M25, P26, Q27, Y28, D29, V30, T31, P32, E33	11
2A		
2C		
1	V320, G321, L324, E325, A326, L327, F328, Q329	8
2	A204, A205, L206, E207, E208	5
3C		
1	N126, G128, G129	3
2	G1, P2, E5, T154	4

### Conservancy evaluation

The conservancy evaluation was analyzed by CLUSTALW and viewed by ESPript. We submitted the VP1, VP2, VP3, 2A, 2C, and 3C amino-acid sequences of different serotypes of CVB to perform multiple sequence alignment (**Figure S1**). Epitopes were selected for vaccine construction only if they were in highly conserved regions. Finally, 11 CTL epitopes, 5 HTL epitopes, and 13 linear and 5 discontinuous B-cell epitopes were considered as conserved epitopes and selected for vaccine construction (**Table 6**).

**Table 6:** Highly conserved epitopes used for the final vaccine construction.

Protein	CTL epitopes	HTL epitopes	Linear B-cell epitopes	Conformational B-cell epitopes
VP1				
1	ILTHQIMYV		HVKAWIPRPPRL	
2	APPRMSIPF		TSTNPSVFWTE	
VP2				
1	IVMPYTNSV	LRTNNSATIVMPYN	EDQPTQPDVATC	
2			SATIVMPYTNSVP	
VP3				
1	YTHWSGSIK	EILNYYTHWSGSIKLTF	DDFQSPSAMPQYDVT	SAMPQYDVTPE
2A				
1	AVYVGNYRV	EGVVGFADIRDLLWL	VTMGGEGVVG	
2	YPRRYQSHV		AGFSEPGDCGGILRC	
2C				
1	KLNSSVYSL	SVATNLIGRSLAEKL	ASTNAGSINAP	VGLEALFQ
2	KMSNYIQFK	ALARRFHFDMNIEVI	HGSPGAGKSV	AALEE
3	QVRYSLDML		TIEQSAPSQSDQEQL	
4			PPDPDHFDGYKQQ	
3C				
1	AVLAINTSK		MYNFPTRAGQCG	NGG
2	RAGQCGGVL		HVGGNGHQGFSAA	GPET

### Multi-epitope vaccine construction and secondary structure prediction

**Figure 2:**
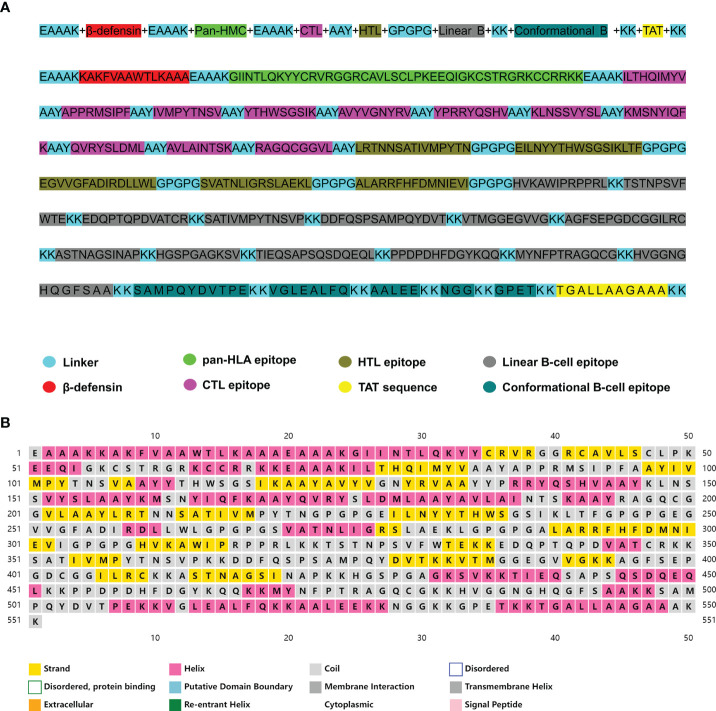
**(A)** The complete sequence of the coxsackievirus B (CVB) vaccine. **(B)** Prediction of the secondary structure of the multi-epitope vaccine construct.

The vaccine was further constructed using the most favorable candidate epitopes. The CTL epitopes, HTL epitopes, and linear and discontinuous B-cell epitopes were joined together with the help of a suitable linker. To promote immunogenicity, the human β-defensin-3 sequence and pan-HLA DR binding epitopes were attached to the N-terminal of the vaccine with the help of the EAAAK linker. Subsequently, the epitopes of CTL, HTL, and B cell derived from CVB were merged by the AAY, GPGPG, and KK linkers, respectively. Also, a TAT sequence was appended to the C-terminal to ensure the intracellular delivery of the modeled vaccine. The final vaccine consists of 551 amino acids (**Figure 2A**). The Blastp analysis confirmed that the protein sequence of the constructed vaccine is non-homologous against the human protein sequence (**Figure S2**).

The secondary structure of the vaccine was predicted *via* the PSIPRED online server, which predicted the secondary structure according to the amino acid sequence of the protein. The results indicated that the secondary structure of the designed vaccine contained 31.03% (171 aa) α-helix, 21.05% (116 aa) β-sheet, and 47.92% (264 aa) random coil (**Figure 2B**).

### Allergenicity, antigenicity, and solubility evaluation

The AllerTOP and AllergenFP online servers were employed to evaluate the allergenicity of the final vaccine. The results showed that the vaccine sequence on these two servers is defined as non-allergen in nature. The antigenicity value of the designed vaccine was calculated *via* the VaxiJen v2.0 server followed by the ANTIGENPro server. VaxiJen predicted the antigenicity score of the vaccine design to be 0.6791 with the bacteria model by default a threshold of 0.4. ANTIGENPro predicted the antigenicity score of 0.9239. Both results clearly revealed that the novel CVB vaccine is an excellent antigen. The SolPro and Protein-Sol servers were utilized to evaluate the solubility of the designed vaccine and its subunit. The results revealed that the final vaccine and each of its subunits have good solubility (**Table 7**).

**Table 7:** Evaluation of the antigenicity, allergenicity, and solubility of the vaccine.

Subunits	VaxiJen score	ANTIGENPro score	AllerTOP result	AllergenFP result	SolPro score	Protein-Sol score
HTL epitope	0.5178	0.4085	Non-allergen	Non-allergen	0.5683	0.4590
CTL epitope	0.4877	0.6310	Non-allergen	Non-allergen	0.6090	0.6670
Linear B-cell epitope	0.4538	0.7101	Non-allergen	Non-allergen	0.9455	0.8060
Conformational B-cell epitope	0.7267	0.8013	Non-allergen	Non-allergen	0.6274	0.9430
Final vaccine	0.6719	0.9239	Non-allergen	Non-allergen	0.9386	0.4840

### Toxicity and physicochemical parameter evaluation

The ToxinPred2 server predicted that the final vaccine construction was non-toxin. In addition, the final vaccine constituted 551 amino acids, and its molecular weight was estimated to be 59.4 kDa. The theoretical p*I* (protein isoelectric point) value was calculated to be 9.84. There were 35 negatively charged residues (Asp + Glu) and 83 positively charged residues (Arg + Lys) in the vaccine. The vaccine construction consisted of 8,377 atoms, and its chemical formula was C_2657_H_4196_N_746_O_755_S_23_. The instability index (II) was 34.59 < 40, suggesting the vaccine to be a stable protein. The estimated half-life was 1 h in mammalian reticulocytes *in vitro*. *In vivo*, the estimated half-life in yeast was about 30 min and more than 10 h in *E. coli*. The aliphatic index of the designed vaccine was 66.01 indicating thermos ability. The GRAVY was found to be −0.443, which suggests a hydrophilic nature of the vaccine. The toxicity and physicochemical parameters of the HTL, CTL, and B-cell epitopes were also assessed, and the results are shown in **Table 8**.

**Table 8:** Evaluation of the toxicity and physiochemical parameters of the vaccine.

Subunits	Toxicity	PI	Half-life (*in vitro*)	Half-life (*in vivo*)	Aliphatic index	Instability index	Molecular weight
HTL epitope	Non-toxin	6.93	5.5 h	2 min	85.10	29.41	10,629.12
CTL epitope	Non-toxin	9.66	1 h	>10 h	85.98	32.70	14,727.00
Linear B-cell epitope	Non-toxin	9.84	3.5 h	>10 h	40.26	43.04	20,466.27
Conformational B-cell epitope	Non-toxin	9.57	1.9 h	>10 h	52.44	34.53	4,503.24
Final vaccine	Non-toxin	9.84	1 h	>10 h	66.01	34.59	59,408.47

### Characterization of the immune response profile of the vaccine

**Figure 3:**
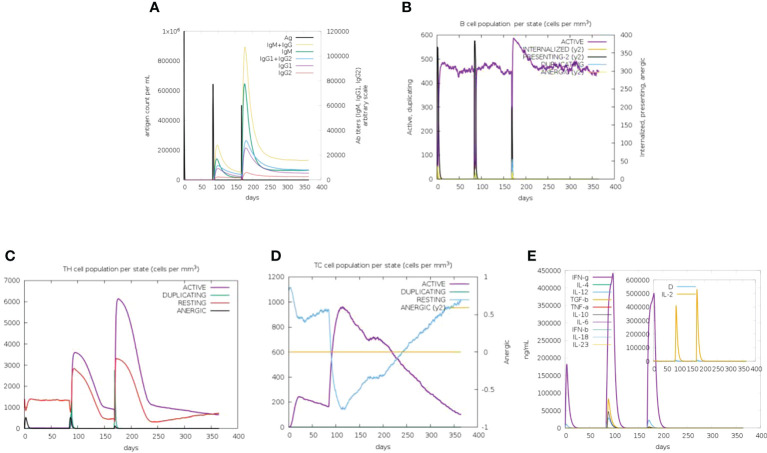
*In-silico* immune simulation of the CVB vaccine by the C-ImmSim tool. **(A)** Antibody production in response to antigen injections (vertical black lines). **(B)** Level of B-cell population. **(C)** Level of helper T-cell population. **(D)** Level of cytotoxic T-cell population. **(E)** Level of cytokine expression.

To evaluate the immune response of the final vaccine, the C-ImmSim online server generated such simulations that are consistent with the real response reactions formed by the immune system. After immunization, the host immune response was obviously activated (**Figure 3**). As shown in **Figure 3A**, the level of the secondary and tertiary antibodies (IgM and IgG) was significantly higher than that of the primary antibodies. Moreover, B-cell, cytotoxic T-cell, and helper T-cell populations were increased significantly (**Figures 3B–D**). The production of various cytokines was also observed after immunization (**Figure 3E**). These results confirm that the vaccine could induce a robust immune response against CVB.

### Prediction, refinement, and quality assessment of the tertiary structure of the developed vaccine construct

**Figure 4:**
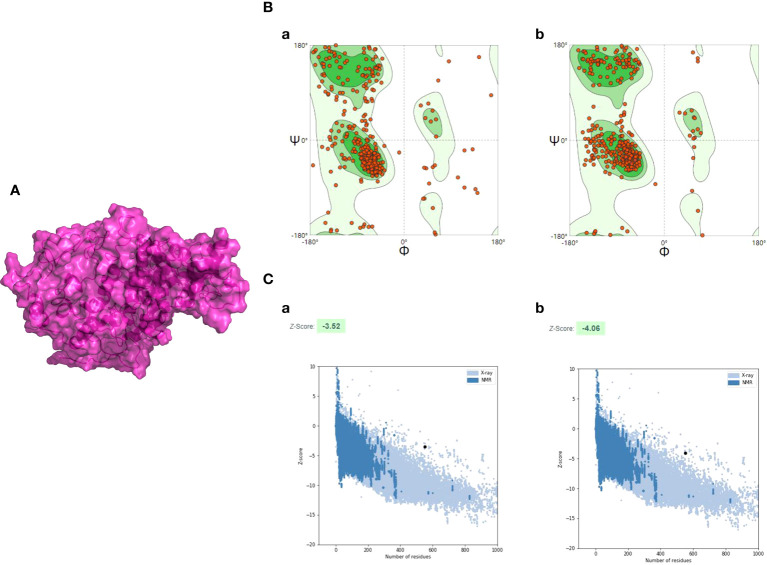
The refinement and quality assessment of the CVB vaccine construct. **(A)** The refinement model of the CVB vaccine construct. **(B)** (a) The Ramachandran plot of the primary model and (b) the Ramachandran plot of the refined model. **(C)** (a) The *Z*-score of the initial model was −3.52, and (b) the *Z*-score of the refined model is −4.06; both models were not in the range of native protein conformation. The *Z*-score plot contains *Z*-scores of all experimental protein chains in PDB determined by NMR spectroscopy (dark blue) and X-ray crystallography (light blue).

The 3D structure of the multi-epitope vaccine was predicted by the AlphaFold2 program, and then it was applied for model refinement and further evaluation. For each prediction, AlphaFold2 could provide five different 3D structural models, and the highest-ranking structure with the best prediction score was selected for further analysis (**Figure S3**). To improve the quality of the initial model, a 50-ns dynamic simulation was performed, and the trajectory generated by the MD was cluster analyzed using the CPPTRAJ program. After clustering, the CPPTRAJ program provided five different clusters. Cluster 1 contained the most frames and was considered the most stable structure, so the representative structure of this cluster was selected as the final structure (**Figure 4A**). Moreover, the Ramachandran plot produced by the SWISS-MODEL tool indicated that the amino-acid residues in the Ramachandran favored regions of the refined model and the original model (95.08% and 77.60%, respectively) (**Figure 4B**).

The ProSA-web server was employed to evaluate the quality and potential errors in the final vaccine 3D model. The quality of the model was reflected by *Z*-score, and generally, the model with a lower *Z*-score has higher quality. The *Z*-score of the original model and the refined model was estimated to be −3.52 and −4.06, respectively (**Figure 4C**).

### Molecular docking of the CVB vaccine with the related antigenic recognition receptor

**Figure 5:**
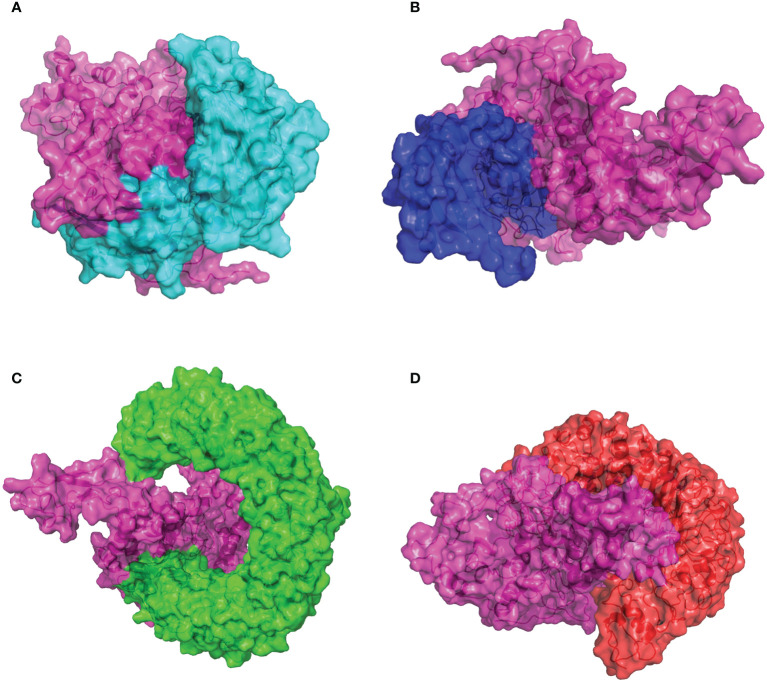
Molecular docking of the CVB vaccine (pink color) with human immune receptors. **(A)** CVB vaccine and MHC-I. **(B)** CVB vaccine and MHC-II. **(C)** CVB vaccine and TLR3. **(D)** CVB vaccine and TLR4.

The capacity of recognition and interaction between the antigenic molecule with the specific immune receptor molecule is necessary to initiate the host immune response. To investigate the binding affinity between the designed CVB vaccine and the relative antigenic receptors (MHC-I, MHC-II, TLR3, and TLR4), the ClusPro2.0 online server was used to perform protein–protein docking. Twenty-five model complexes of each docking were generated, while only the best complex with the lowest binding energy was chosen for further analysis. The complex consisting of the MHC-I and the CVB vaccine with the lowest binding energy score of −1,145.1 kJ/mol was selected to be shown (**Figure 5A**). Evaluating the complex model of the vaccine and MHC-I indicated that the interface area was 1,776.8 Å^2^ and 19 hydrogen bonds were formed with the MHC-I residues (**Table S1**). The complex consisting of the MHC-II and the vaccine with the lowest binding energy score of −1,250.6 kJ/mol was selected to be shown (**Figure 5B**). Evaluating the complex model of the vaccine and MHC-II indicated that the interface area was 1,073.5 Å^2^ and 16 hydrogen bonds were formed with the MHC-II residues (**Table S1**). The complex consisting of the TLR3 and the vaccine with the lowest binding energy score of −1,126.8 kJ/mol was selected to be shown (**Figure 5C**). Evaluating the complex model of the vaccine and TLR3 reflected that the interface area was 1,302.5 Å^2^ and 8 hydrogen bonds were formed with the TLR3 residues (**Table S1**). The complex consisting of the TLR4 and the vaccine with the lowest binding energy score of −1,151.8 kJ/mol was selected to be shown (**Figure 5D**). Evaluating the complex model of the vaccine and TLR4 suggested that the interface area was 2,025.9 Å^2^ and 21 hydrogen bonds were formed with the TLR4 residues (**Table S1**). The hydrogen bond interaction diagrams of each complex are illustrated in **Figure S4**. These protein docking results indicated a strong binding of the vaccine to immune receptors, which means that the designed vaccine could be considered a potential vaccine candidate.

### Molecular dynamics simulation

**Figure 6:**
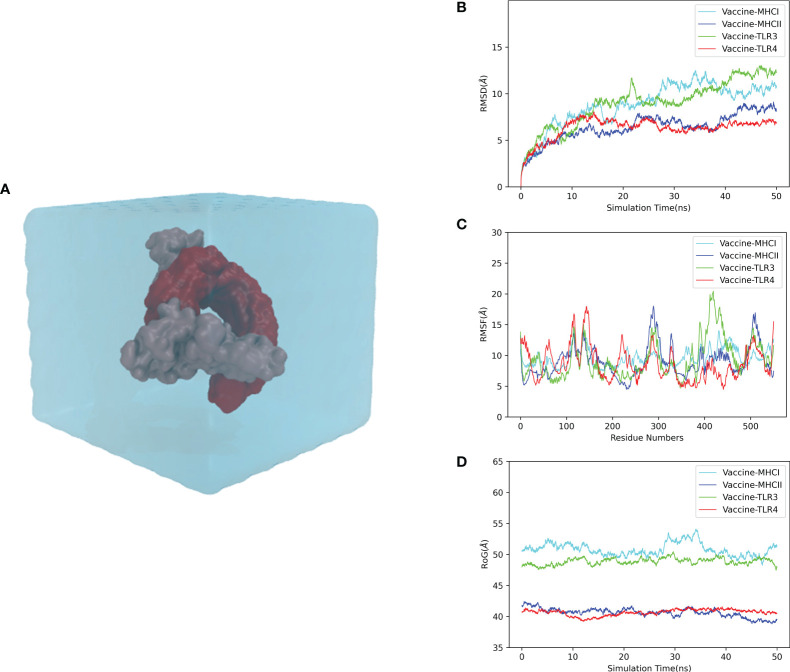
Molecular dynamics simulation of the CVB vaccine–immune receptor complex at 50 ns. **(A)** Molecular dynamics system (prepared for the simulation); **(B)** RMSD (root mean square deviation) of the docked complex reflects the stability between the vaccine. **(C)** RMSF (root mean square fluctuation) reflects the flexibility and fluctuation of the amino-acid residues in the side chain of docked complexes. **(D)** Time evolution of the radius of gyration (rRyr) during 50 ns of MD simulation.

To elucidate the stability and dynamics of the docked complexes, we performed the MD simulation of each complex for 50 ns at 300 K and 1 atmosphere (**Figure 6A**). The simulation results were reflected by the root mean square deviation (RMSD), root mean square fluctuation (RMSF), and radius of gyration (rGyr). As a measure of the structural fluctuation between complexes, RMSD was employed to analyze the stability of the vaccine in the binding domain of the immune receptor. The RMSDs of the vaccine–MHC-I, vaccine–MHC-II, vaccine–TLR3, and vaccine–TLR4 complexes showed a great fluctuation from the beginning of the simulation. After 30 ns, the RMSDs of the vaccine–MHC-I, vaccine–MHC-II, vaccine–TLR3, and vaccine–TLR4 complexes were steady, and the mean RMSD values for the complexes were 10.71, 7.42, 11.08, and 6.52 Å, respectively, suggesting that the conformation of the complexes was stable (**Figure 6B**). The RMSF value reflects the residual flexibility of the docked complex. The RMSF results revealed that residues 350–400 of the vaccine–MHC-I, vaccine–MHC-II, vaccine–TLR3, and vaccine–TLR4 complexes have low RMSF values, indicating that these residues had less variability and were more stable. By contrast, residues 100–170, 250–300, and 400–450 had relatively higher RMSF values, suggesting that these regions fluctuated significantly and had higher flexibility (**Figure 6C**). Moreover, rGyr was employed to evaluate the binding stability of the docked complex. The lower the rGyr value, the more compact the complex, with high folded stability. The average rGyr values detected from the vaccine–MHC-I, vaccine–MHC-II, vaccine–TLR3, and vaccine–TLR4 complexes were 50.78, 40.60, 48.86, and 40.64 Å, respectively (**Figure 6D**).

The binding free energies between the vaccine and the receptor complexes (MHC-I, MHC-II, TLR3, and TLR4) were calculated *via* the MMPBSA method. As shown in **Table 9**, the total energy values of the vaccine–MHC-I, vaccine–MHC-II, vaccine–TLR3, and vaccine–TLR4 complexes were −8.65, −31.36, −43.10, and −9.19 kcal/mol, respectively. Moreover, the calculated values revealed that the electrostatic energy and the van der Waals energy are major contributors to binding free energies. As for the solvation term, while the polar solvation term is negative for binding, the non-polar solvation term has a negligible impact on binding.

**Table 9:** The binding free energy (kcal/mol) of the vaccine and receptor complex.

Energy components	Binding free energies (kcal/mol)			
	Vaccine–MHC-I	Vaccine–MHC-II	Vaccine–TLR3	Vaccine–TLR4
Van der waals	−157.07	−229.96	−138.50	−197.16
Electrostatic energy	−3,064.98	−6,116.41	−2,898.84	−6,857.22
Solvation term	3,234.28	6,347.10	3,012.70	7,072.07
Non-polar solvation	−21.00	−32.06	−18.45	−27.04
Total energy	−8.65	−31.36	−43.10	−9.19

### Codon adaptation, mRNA secondary structure prediction, and *in-silico* cloning

To ensure effective expression in *E. coli* (strain K12), the final vaccine protein sequence was reverse-translated and codon-optimized using the Java Codon Adaptation tool. The optimized codon sequence had a length of 1,653 nucleotides. The restriction sites *Hin*dIII and *Bam*HI were added to the N- and C-ends of the codon sequence and then cloned into the pET28a(+) vector between the *Hin*dIII and *Bam*HI using the SnapGene software (**Figure S5**). The RNA secondary structure was generated *via* the RNAfold program, as illustrated in **Figure S6**.

## Discussion

Vaccine is the most effective way for preventing pathogenic infection, especially viruses. A successful vaccine could induce humoral and cellular immune responses to a specific pathogen and significantly reduce the morbidity and mortality caused by the infection. The conventional vaccine development methods are time-consuming and expensive with a higher chance of failure (81, 82). Immunoinformatics emerges as an optional approach that facilitates designing vaccines with specificity and stability efficiently (83). It is based on the nucleotide or amino acid sequences of the pathogen, using computer-aided immunoinformatics analysis and other techniques to identify B-cell and T-cell epitopes and then construct a multi-epitope vaccine containing dominant epitopes. With revolutionary advances in information technology and sequencing technology, a growing number of researchers employed immunoinformatics approaches to design epitope-based vaccines (27–31, 78). Currently, CVB is regarded as the predominant pathogen of human viral myocarditis, without a clinically available vaccine (4, 84). Hence, this study aimed to develop a novel CVB multi-epitope vaccine with high effectiveness and no side effects using immunoinformatics tools.

The proper selection of protein antigens is essential to design a scientific and rational epitope vaccine. Here, we retrieved the CVB protein components and used them for antigenicity prediction. The proteins with <100 amino-acid sequences were removed, and the antigenic scores of the remaining proteins were calculated through the VaxiJen and ANTIGENPro servers with the threshold of 0.4 (28). Antigenic scores below this threshold were defined as non-antigen and then excluded. This method could facilitate the detection of potential antigens of CVB and avoid adverse factors when clear immunity mechanisms are discovered. Among these high antigenicity proteins, VP1, VP2, and VP3 are structural proteins exposed at the virion surface and rich in T-cell epitopes and neutralizing epitopes, which could induce extensive cellular and humoral immune responses (11). In addition, non-structural proteins are highly conserved and play very important roles during viral replication. Proteins 2A and 3C are viral-encoded proteinases with enzymatic activity that not only cleave virus polyproteins but also inhibit host cell transcription and translation by directly cleaving cell proteins (85, 86). Protein 2C is a membrane protein with helicase activity, which can bind to the cell membrane and affect its permeability. When virus infection causes cell rupture, these proteins will be released and caused damage to other host cells. Cellular and humoral immune responses induced by 2A, 2C, and 3C could effectively protect the host. Therefore, we chose VP1, VP2, VP3, 2A, 2C, and 3C as the target proteins for epitope prediction.

The multi-epitope vaccine composed of B-cell, CTL, and HTL epitopes can trigger extensive immune protection (87, 88). B-cell epitopes, consisting of linear epitopes and conformational epitopes, are antigenic determinants from the surface of antigens that are recognized and bound by B-cell receptors (BCR), which induce a humoral immune response (89). Accurately identifying epitopes from antigen sequences alone is a challenge. In this study, we identify 28 linear B-cell epitopes and 9 discontinuous B-cell epitopes from the antigenic proteins by using classical immunoinformatics tools. T cell, as an important component and effector cell in the immune system, plays an indispensable role in controlling and inducing the immune response (90). T-cell epitopes are composed of peptide fragments from protein antigens presented by MHC molecules of antigen-presenting cells and stimulated the generation of effector T cells, immunological memory T cells, and cytokines (such as IFN-γ). The specific cellular immune response induced by CTLs plays a significant role in eliminating viruses and infected cells through the recognition of intracellular viral pathogens by MHC class I molecules (91). To overcome the polymorphism of HLA, we used A2, A3, and B7 HLA alleles to predict MHC-I binding epitopes. Those alleles are representative of HLA supertypes, and at least 95% of the world’s population expresses an allele included in these supertypes. A total of 17 CTL epitopes were selected. The HTLs play an important role in the antiviral immune response by secreting IFN-γ. In addition, HTLs can promote and support the expansion and differentiation of CTL and B-cell precursors into effector cells. To achieve more population coverage, HTL epitopes were predicted by choosing several HLA alleles. A total of 13 HTL epitopes were selected according to the capability of binding affinity and IFN-γ stimulation. Since picornaviruses such as CVB have extremely high mutability, to ensure the stability of the epitopes selected in this study, we performed multiple sequence alignment of CVB between different serotypes, and only the epitopes located in the highly conserved region were selected. Finally, 11 CTL epitopes, 5 HTL epitopes, and 13 linear and 5 conformational B-cell epitopes were selected for the final vaccine construction.

The multi-epitope vaccine was constructed by splicing the B-cell, CTL, and HTL epitopes with KK, AAY, and GPGPG linkers, respectively. Linkers are an essential element of vaccines to ensure that each epitope can independently trigger the immune response and also avoid the creation of a new epitope that interferes with the immune response induced by the original epitope (92). The immunogenicity of multi-epitope vaccines is poor when used alone and adjuvant coupling is required (93). Adjuvants are important components in vaccine formulations that protect against infection and influence the specific immune responses, growth, stability, and persistence of the antigens (94). Therefore, to improve the immunogenicity of this vaccine, the adjuvant β-defensin and pan-HLA DR binding epitopes were fused to the N-terminal with the help of the EAAAK linker, then a TAT sequence was appended to the C-terminal by the KK linker. The final vaccine stretch with the addition of adjuvant and linkers was found to be 551 amino acids long. The secondary structure of the multi-epitope vaccine revealed that the random-coil dominated the structure, indicating that the vaccine structure is relatively loose and easy to twist and protrudes outward, which is conducive to chimerism with the antibody.

Next, the physicochemical characteristics of the designed vaccine were analyzed. The molecular weight of the vaccine construct was 59.4 kDa, and the theoretical p*I* was 9.84, which showed the basic nature of the vaccine construct. The instability index, GRAVY value, and aliphatic score suggested that the vaccine protein is stable, hydrophobic, and thermostable. The evaluation of allergenicity and antigenicity indicated that the vaccine is immunogenic, strongly antigenic, and non-allergenic. Furthermore, the immune stimulation indicated that the vaccine could promote the expansion and differentiation of B- and T-cell precursors into effector cells and trigger high levels of IgG, IgM, and cytokines. These results suggested that the designed multi-epitope vaccine can elicit a robust immune response without allergic reactions.

The 3D structure provides the spatial coordinate information of all atoms in protein molecules and lays the foundation for subsequent research on ligand interactions, protein functions, and dynamic simulation (95, 96). In this study, the 3D structure of the CVB vaccine was generated by using the AlphaFold2 program, and then MD simulation was performed to refine the initial model. After refinement, the quality of the initial modeled structure has been greatly improved. The Ramachandran plot demonstrated that the great majority of residues are distributed in favored or additional allowed regions, while only a small minority of residues were located in the disallowed region, which implies an excellent quality of the refined model. Additionally, the refined model structure was queried for potential errors *via* the ProSA-web server. The result showed that the *Z*-score was −4.06 proving that the overall structure of the refined model is reliable and of good quality.

A strong interaction between the antigenic molecule and the immune receptor molecule (MHC-I, MHC-II, TLR3, and TLR4) is necessary to initiate the immune response (97, 98). Molecular docking and molecular dynamics simulation not only proved the stable interactions between the refined vaccine construct and the immune receptor but also revealed that electrostatic energy and van der Waals energy are major contributors to this proficient binding. Several hydrogen bonds were observed during the vaccine construct docked against immune receptors, and quite minor fluctuations were observed during the molecular dynamics simulation. These results clearly revealed that the developed vaccine can perfectly bind to the immune receptors, and therefore, the developed multi-epitope vaccine might be able to induce a robust immune response. Interestingly, based on previous studies (99, 100), some monoclonal antibodies against group A streptococci identify cross-reactive epitopes in cardiac tissues and can also neutralize the myocardial pathogenicity of CVB3 (H3). The T lymphocytes from CVB3 (H3)-infected mice are responsive to certain peptides from the streptococcal M protein. These observations suggest that our multi-epitope vaccine may have the potential of protecting from cardiac injury caused by group A streptococcus. It is worthy of validation with animal experiments.

Moreover, to ensure the efficient expression of the CVB vaccine protein in the *E. coli* system, the amino acid sequence of the vaccine was reverse-translated and codon-optimized by utilizing the Java Codon Adaptation tool. Then, *Hin*dIII and *Bam*HI restriction sites were added to the 5′ and 3′ ends of the codon sequence. The final vaccine sequence was subsequently cloned into the pET28a(+) vector.

As the research is presently based on *in-silico* prediction, this may raise questions that the epitopes presented by the supertype MHC molecules can generate virus-reactive but not protective immunity (101–103). Only a few out of the miscellaneous antibodies induced by viruses are viral neutralizing. It is impossible to judge if this approach would be beneficial to the human population without functional evaluation of the immunity induced by the putative vaccine. Therefore, further *in-vivo* experiments are necessary to validate the safety and efficacy of the designed vaccine. However, since the vaccines are based on human HLA, functional evaluation is actually difficult to be carried out using routine experimental animals. Even though there are humanized mice, it is still recognized that testing the vaccine in such a model would be difficult (104, 105). Nonetheless, it is possible to determine *in vitro* if the antibodies generated were neutralizing and to determine the cytolytic potential of the T cells (106, 107). We will check it later as an independent study.

In conclusion, this research constructed a multi-epitope vaccine based on the highly conserved epitopes among different serotypes of CVB, which can effectively solve the problem that currently designed vaccines can only provide effective immune protection against a single serotype CVB (15–19, 108). Further analysis indicated that the designed vaccine was highly antigenic and non-toxic and could induce robust, multiple serotype-specific immune responses, so it has preferable practicability. In addition, the vaccine structure can stably bind to the human immune recognition receptor, triggering a persistently and strong immune response. This study opens the way for the development of a multi-epitope CVB vaccine and also provides a convenient and systematic approach for researchers to design an epitope vaccine against other pathogens with multi-serotypes.

## Data availability statement

The datasets presented in this study can be found in online repositories. The names of the repository/repositories and accession number(s) can be found below: https://www.ncbi.nlm.nih.gov/, AAA42931.1; http://www.wwpdb.org/, 2A0Z; http://www.wwpdb.org/, 4WUU; http://www.wwpdb.org/, 3C5J; https://www.ncbi.nlm.nih.gov/, AAC00531.1; https://www.ncbi.nlm.nih.gov/, AUF49670.1; https://www.ncbi.nlm.nih.gov/, AAL37156.1; https://www.ncbi.nlm.nih.gov/, QAT18823.1; https://www.ncbi.nlm.nih.gov/, ALA40024.1; http://www.wwpdb.org/, 3FXI.

## Author contributions

SH and YW conceived the study. SH, Z-HZ and YW designed the experiment. SH and CZ performed the computational analysis. JL, ZD, JH, FD and XW collected the data. SH wrote the manuscript. XY, XH, YL, YD and YHW provided valuable suggestions to improve the manuscript. WZ and Z-HZ provided professional consulting support. All authors contributed to the article and approved the submitted version.

## Funding

This work was supported by the National Natural Science Foundation of China (grant nos. 81772188, 81571999, 81871652, and 82072278).

## Acknowledgments

We thanked PhD. Lianhao Song for assisting us in molecular dynamics simulation analysis.

## Conflict of interest

The authors declare that the research was conducted in the absence of any commercial or financial relationships that could be construed as a potential conflict of interest.

## Publisher’s note

All claims expressed in this article are solely those of the authors and do not necessarily represent those of their affiliated organizations, or those of the publisher, the editors and the reviewers. Any product that may be evaluated in this article, or claim that may be made by its manufacturer, is not guaranteed or endorsed by the publisher.

## Supplementary material

Figure S1 The multiple sequence alignment between different serotypes of CVB, the epitope selected for vaccine construct have been identified by black boxes. **(A)** Multiple sequence alignment of VP1 protein sequences. **(B)** Multiple sequence alignment of VP2 protein sequences. **(C)** Multiple sequence alignment of VP3 protein sequences. **(D)** Multiple sequence alignment of 2A protein sequences. **(E)** Multiple sequence alignment of 2C protein sequences. **(F)** Multiple sequence alignment of 3C protein sequences.

Figure S2 The analysis of the sequence homology of the CVB vaccine construct.

Figure S3 The original 3D structure model of the CVB vaccine prediction by the AlphaFold2 program.

Figure S4 Analysis the interaction between the CVB vaccine and immune receptors (Salt bridges colored in rad); Disulphide bonds colored in yellow; Hydrogen bonds colored in blue; Non-bounder contacts colored in orange. **(A)** CVB vaccine and MHC-I. **(B)** CVB vaccine and MHC-II. **(C)** CVB vaccine and TLR3. **(D)** CVB vaccine and TLR4.

Figure S5
*In silico* cloning of the CVB vaccine in the vector, pET28a (+). Red areas represent the CVB vaccine, while the black areas represent the expression vector, pET28a (+).

Figure S6 The secondary structure of the designed vaccine mRNA.
