# Supplementary material for: Designing a multi-epitope vaccine against coxsackievirus B based on immunoinformatics approaches
Source: Front Immunol. 2022 Nov 9;13:933594. doi: 10.3389/fimmu.2022.933594 (PMC9682020; doi:10.3389/fimmu.2022.933594)
Supplement: Supplementary file 1 [file Table_1.docx]

**Table S1 |** Molecular docking of the final vaccine with MHC-I, MHC-II, TLR3, and TLR4.

| **Receptor** | **Center**  **(kJ/mol)** | | **Lowest energy**  **(kJ/mol)** | | **Interface area**  **(Å^2^)** | **Hydrogen**  **bonds** |
| --- | --- | --- | --- | --- | --- | --- |
| MHC-I | -1020.7 | -1145.1 | | 1776.8 | | 19 |
| MHCII | -1181.2 | -1250.6 | | 1073.5 | | 16 |
| TLR3 | -1026.3 | -1126.8 | | 1302.5 | | 8 |
| TLR4 | -1111.9 | -1151.8 | | 2025.9 | | 21 |
