# Supplementary figures and images for: Designing a multi-epitope vaccine against coxsackievirus B based on immunoinformatics approaches
Source: Front Immunol. 2022 Nov 9;13:933594. doi: 10.3389/fimmu.2022.933594 (PMC9682020; doi:10.3389/fimmu.2022.933594)

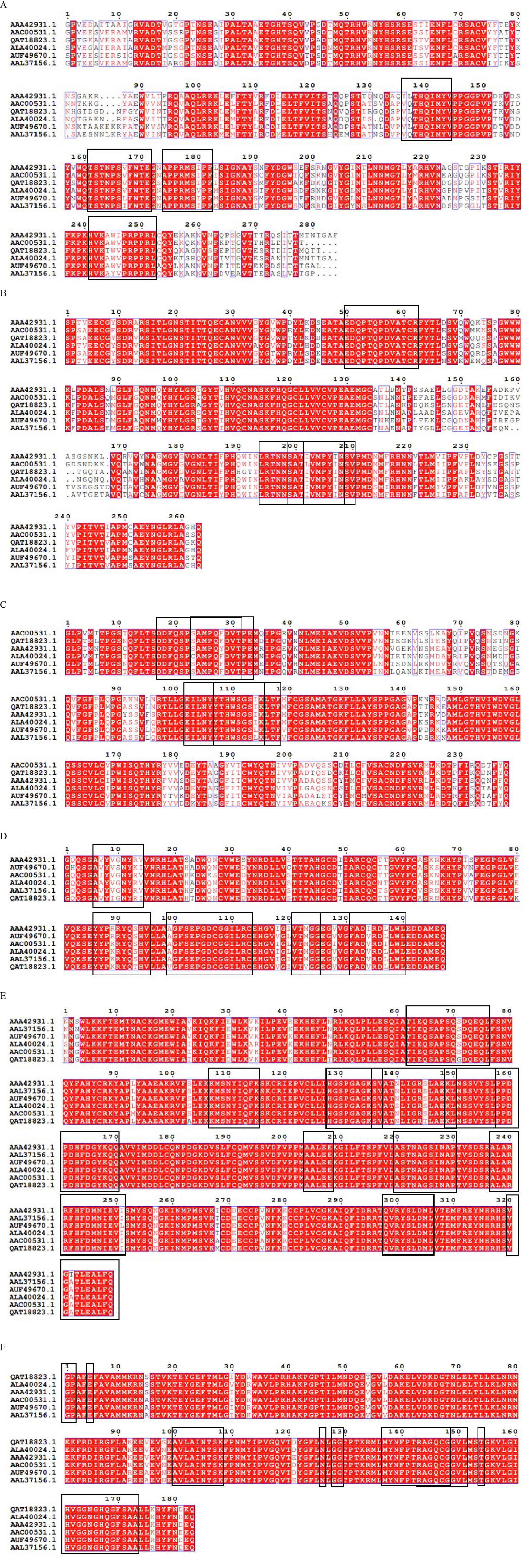

Supplement: Figure S1 — The multiple sequence alignment between different serotypes of CVB, the epitope selected for vaccine construct have been identified by black boxes. (A) Multiple sequence alignment of VP1 protein sequences. (B) Multiple sequence alignment of VP2 protein sequences. (C) Multiple sequence alignment of VP3 protein sequences. (D) Multiple sequence alignment of 2A protein sequences. (E) Multiple sequence alignment of 2C protein sequences. (F) Multiple sequence alignment of 3C protein sequences. [file Image_1.jpg]

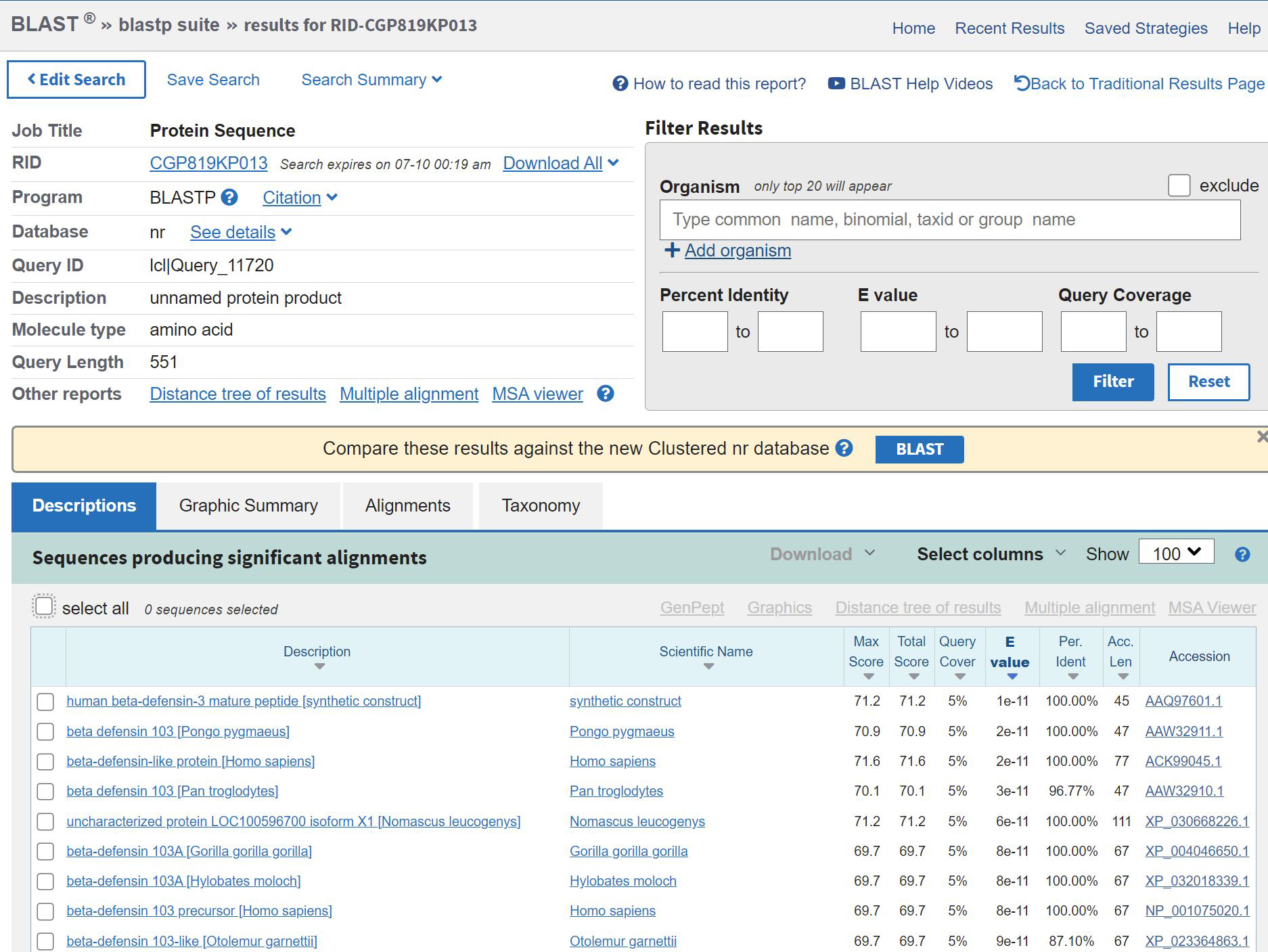

Supplement: Figure S2 — The analysis of the sequence homology of the CVB vaccine construct. [file Image_2.jpeg]

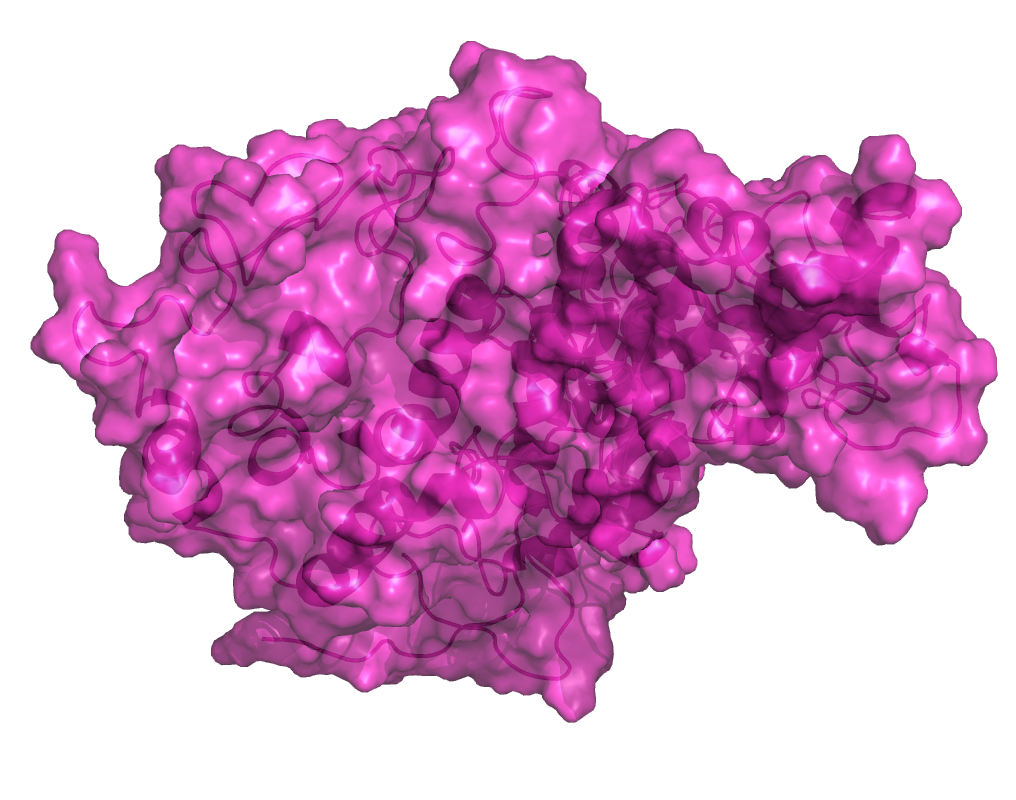

Supplement: Figure S3 — The original 3D structure model of the CVB vaccine prediction by the AlphaFold2 program. [file Image_3.png]

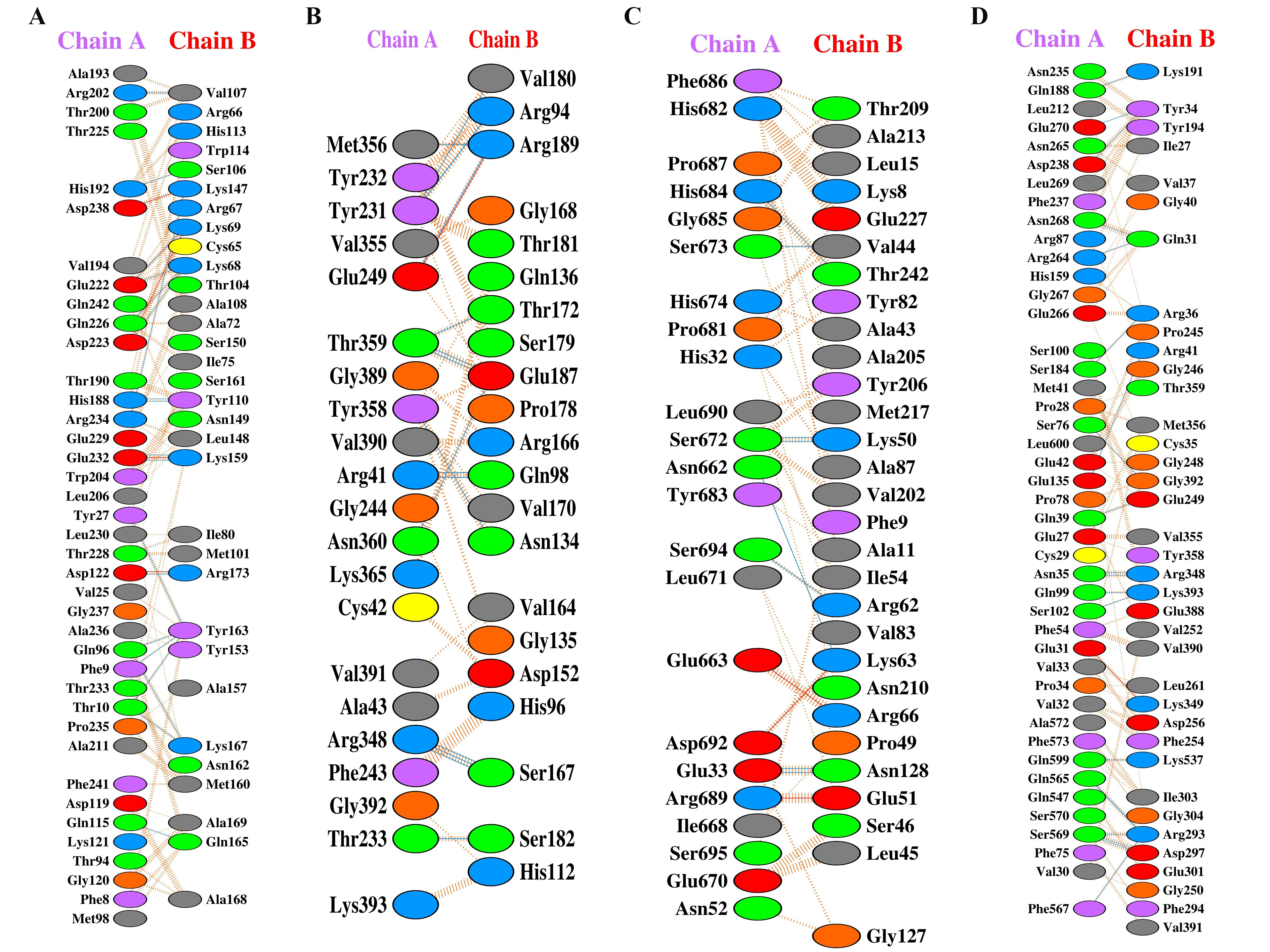

Supplement: Figure S4 — Analysis the interaction between the CVB vaccine and immune receptors (Salt bridges colored in rad); Disulphide bonds colored in yellow; Hydrogen bonds colored in blue; Non-bounder contacts colored in orange. (A) CVB vaccine and MHC-I. (B) CVB vaccine and MHC-II. (C) CVB vaccine and TLR3. (D) CVB vaccine and TLR4. [file Image_4.jpeg]

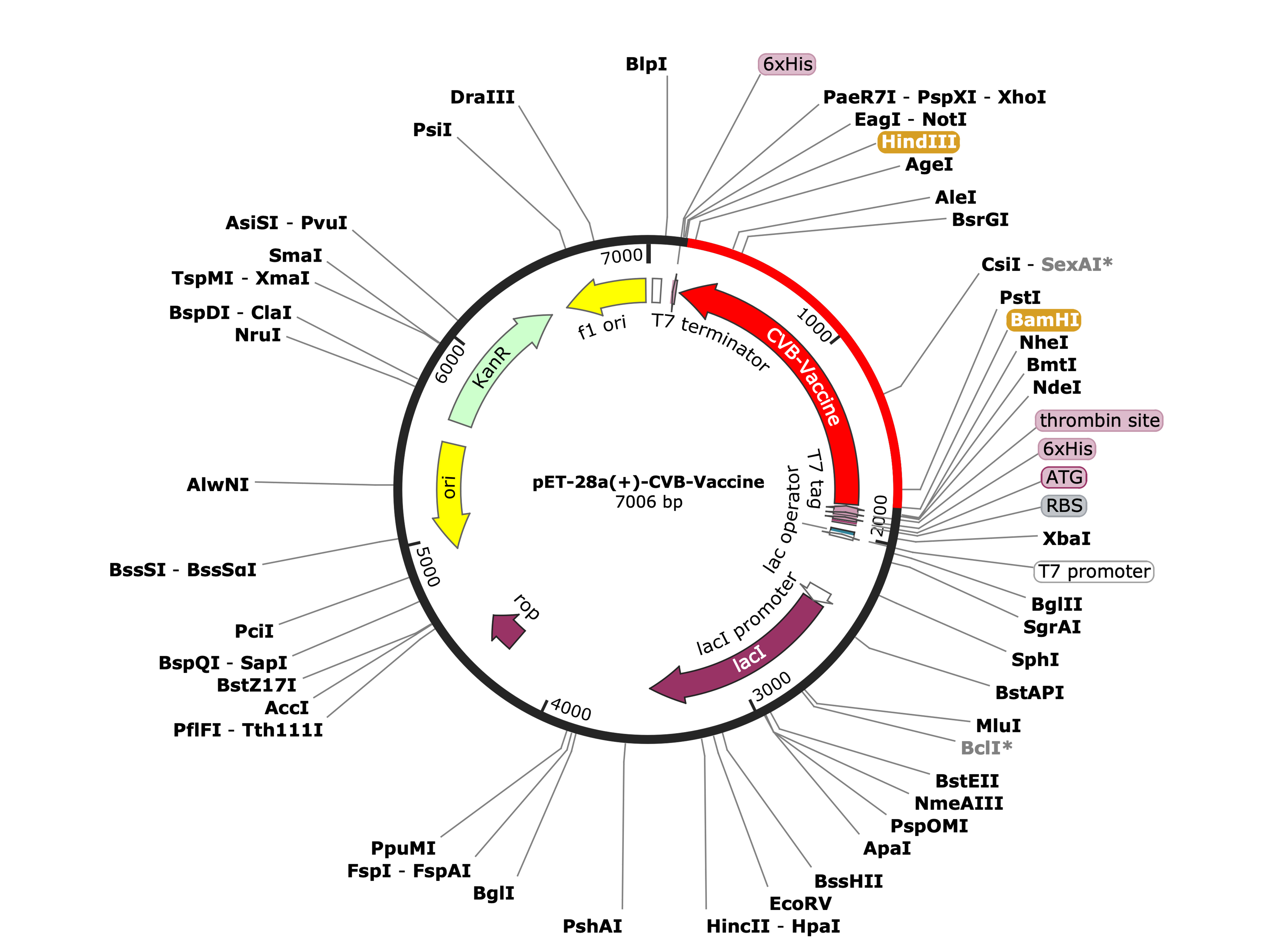

Supplement: Figure S5 — In silico cloning of the CVB vaccine in the vector, pET28a (+). Red areas represent the CVB vaccine, while the black areas represent the expression vector, pET28a (+). [file Image_5.jpeg]

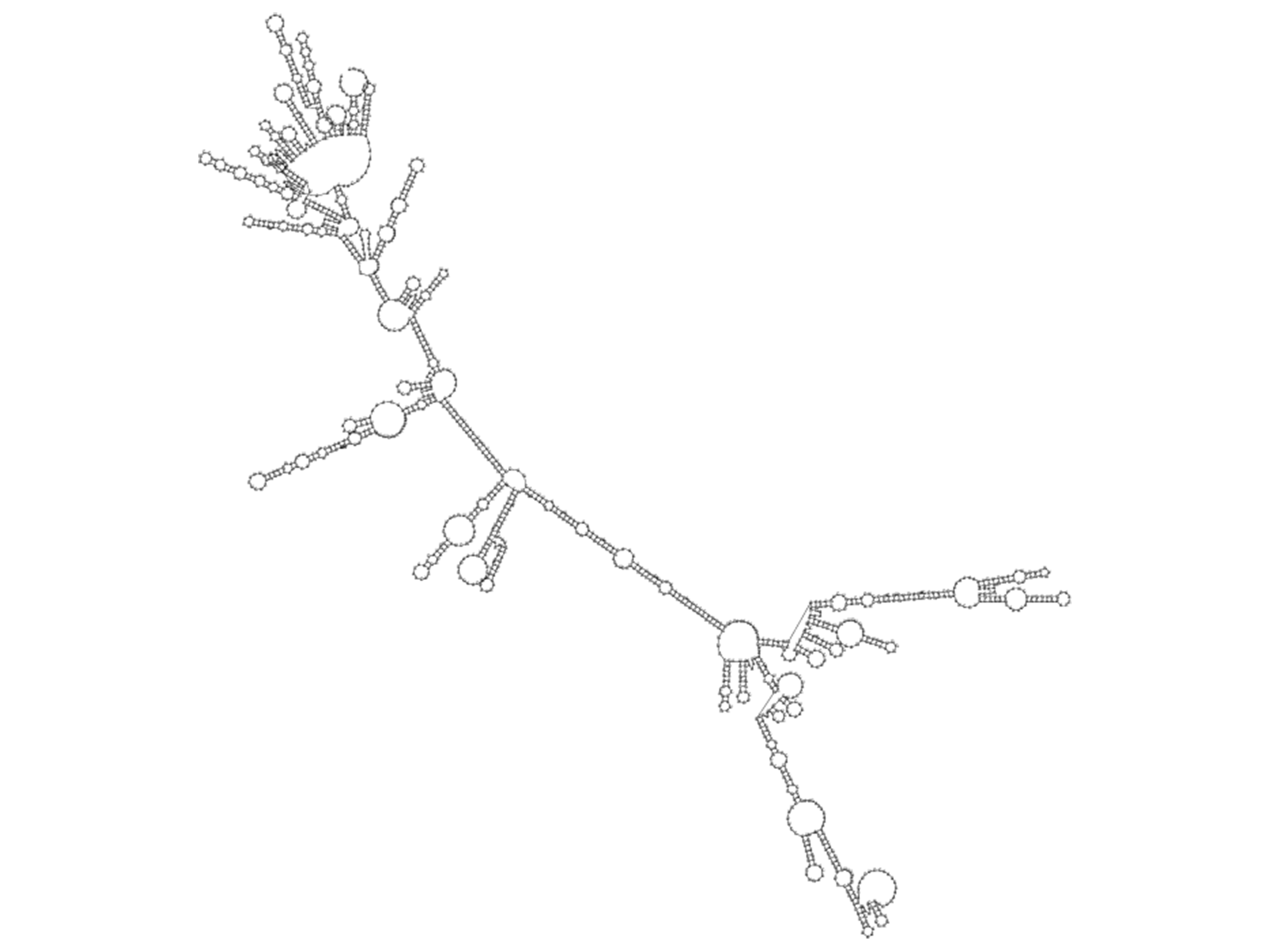

Supplement: Figure S6 — The secondary structure of the designed vaccine mRNA. [file Image_6.jpeg]
